# The Structure of a Gene Co-Expression Network Reveals Biological Functions Underlying eQTLs

**DOI:** 10.1371/journal.pone.0060045

**Published:** 2013-04-05

**Authors:** Nathalie Villa-Vialaneix, Laurence Liaubet, Thibault Laurent, Pierre Cherel, Adrien Gamot, Magali SanCristobal

**Affiliations:** 1 INRA, UR875, Unité de Biométrie et Intelligence Artificielle (UBIA), Castanet Tolosan, France; 2 INRA, UMR444 Laboratoire de Génétique Cellulaire, Castanet Tolosan, France; 3 Toulouse School of Economics, Université Toulouse 1, Toulouse, France; 4 Hendrix Genetics RTC, St. Jean en Braye, France; Wageningen UR Livestock Research, The Netherlands

## Abstract

What are the commonalities between genes, whose expression level is partially controlled by eQTL, especially with regard to biological functions? Moreover, how are these genes related to a phenotype of interest? These issues are particularly difficult to address when the genome annotation is incomplete, as is the case for mammalian species. Moreover, the direct link between gene expression and a phenotype of interest may be weak, and thus difficult to handle. In this framework, the use of a co-expression network has proven useful: it is a robust approach for modeling a complex system of genetic regulations, and to infer knowledge for yet unknown genes. In this article, a case study was conducted with a mammalian species. It showed that the use of a co-expression network based on partial correlation, combined with a relevant clustering of nodes, leads to an enrichment of biological functions of around 83%. Moreover, the use of a spatial statistics approach allowed us to superimpose additional information related to a phenotype; this lead to highlighting specific genes or gene clusters that are related to the network structure and the phenotype. Three main results are worth noting: first, key genes were highlighted as a potential focus for forthcoming biological experiments; second, a set of biological functions, which support a list of genes under partial eQTL control, was set up by an overview of the global structure of the gene expression network; third, pH was found correlated with gene clusters, and then with related biological functions, as a result of a spatial analysis of the network topology.

## Introduction

Integrative and systems biology is a very promising tool for deciphering the biological and genetic mechanisms underlying complex traits. In this context, gene networks are used to model interactions between genes of interest: gene networks have been increasingly applied to understand the basis of complex biological phenomena [1], [2].

A gene network can be variously defined. For instance, some are based on bibliographic knowledge obtained by literature mining with software like Ingenuity Pathway Analysis (IPA), Pathway Studio or Cytoscape (compared in [3], [4]). Others combine experimental and computational approaches to define Protein-Protein Interaction - PPI - networks [5] or known biochemical and physiologic data to define metabolic networks [6]. Although biological knowledge networks are useful tools, they have some limitations due to a major lack of annotation of the genomes, and the fact that most associated literature is devoted mainly to only a few mammalian species (e.g., humans, mice and rats in IPA).

An alternative approach is to infer the network directly from gene expression data, leading to the definition of a so-called “gene co-expression network” [7]. Inferring a co-expression network directly from gene expression data aims at focusing on direct co-expressions between genes by calculating, for instance, partial correlations [8]. Unlike in ontological enrichment analysis or bibliographic networks, information available on both functionally known and unknown genes is used for the network definition.

Once the network is given, a full analysis of its structure could be performed, from either the point of view of the network [9]–[11], or in correlation with a variable of interest [12]. Such analyses search for key genes, or for functional modules, or also for an understanding of the relations between the network structure and additional information (e.g., a phenotype of interest). However, regardless of the increasing number of papers focusing on networks only a few present a full analysis, starting from raw expression data, then inferring and mining the network to end up with an understanding of its relation with an external variable. For instance, [13] demonstrates the usefulness of network inference and mining for the analysis of microarray data: in the present article, the process is pushed further, allowing ones to integrate information pertaining to a phenotype. Similarly, [14] integrates expression data and PPI bibliographic network to identify candidate genes associated with a given phenotype but they do not rely on a network directly based on expression data.

In the present article, a thorough analysis is conducted. In a previous study [15], gene expressions regulated by eQTLs had been identified. 272 genes have been outlined and their biological relevance studied for those that were already annotated. Indeed the limited annotation prevented the performance of functional annotation for each cluster of eQTL. Moreover, the possible interactive links between these 272 genes, whose expression are partially regulated by eQTL, has not yet been investigated. These links can be an insight on the biological processes and can lead to the extraction of particularly important genes that are good candidates for further biological experiments.

Moreover the eQTL analysis has been done without preselecting genes to be related to a phenotype of interest. Therefore the present analysis of the gene co-expression network was made in relation to a complex phenotype, e.g., muscle pH. The muscle pH has a major industrial interest, as it is well known to be related to meat quality [16]. As the expression of the 272 genes regulated by an eQTL is only weakly correlated to muscle pH (these genes were not selected to be differentially expressed), individual analysis of gene correlation with pH is not relevant in our case. Nevertheless, our proposal is to focus on gene clusters rather than on individual relations, because clusters are more robust (i.e., less prone to be modified by noisy measurements) than each individual relation [17]. We also used an approach based on spatial statistics in order to highlight important genes that are related to the muscle pH and also to the network structure.

Focusing on this dataset, the purpose of the present paper is to gain biological knowledge from expression data for a set of genes that are partially controlled by eQTL, by proposing an adequate statistical pipeline. This pipeline is aimed at being a general tool for dissecting biological functions and interactions. The context of this work is a mammalian species with medium to low genome annotation and a gene list that does not result from a differential analysis. The proposed statistical pipeline will be briefly presented, as well as the main results, in the first Section. The Section “Materials and Methods” will then describe it in details.

## Results

**Figure 1:**
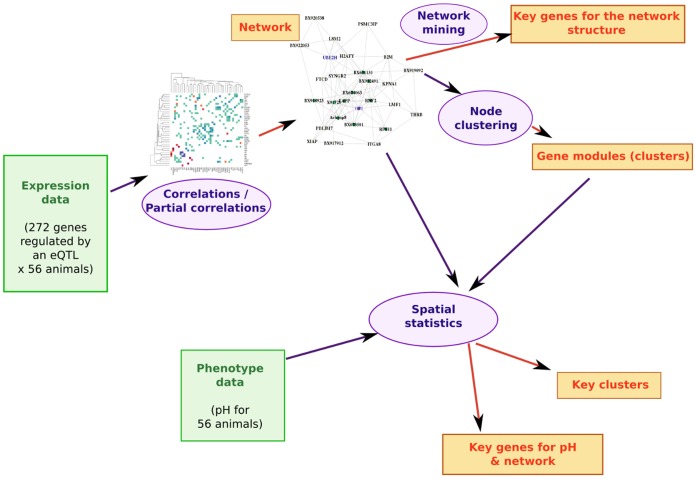
Summary of the statistical pipeline. Data are represented in green (expression data and pH), statistical methods are represented in purple, results are represented in red.

The raw data, which consisted of the expression of 272 genes partially controlled by eQTL, were measured *post mortem* in a muscle on 56 half sibs [15]. The statistical pipeline that was used to gain knowledge from our list of genes is summarized in Figure 1. In the remaining of this section, all results obtained from the statistical analysis are described. The following section discusses these results and a final Section “Materials and Methods” provides further details on the dataset, on the statisical methods and on their validation.

A co-expression network is first built from the 272 gene expressions, and the structure of this network is highlighted, in terms of nodes of particular importance (hubs for instance), and in terms of decomposition into “communities” or “modules”. The biological meaning of each gene or of each set of genes is systematically investigated in order to validate the statistical tools. Finally, the way a quantitative trait is related to the structure of the network, is analyzed.

### Network Definition

A co-expression network between the 272 genes was built on partial correlations using the Gaussian Graphical Model (GGM) approach described in [8]. In this model, the network nodes are the 272 genes and edges between two nodes, which model significant correlations between the expressions of the corresponding genes. To measure the strength of the link between gene expressions, partial correlations were estimated: they are defined as the correlations between the expression of two genes *knowing the expressions of all the other genes*. As pointed out by [13], because networks focus only on the most significant links between genes, they are far less subject to noisy data; as such, they are a more robust approach than conventional analyses based on raw expression data to extract key genes and find groups of highly co-expressed genes. Moreover, the use of a partial correlation based network was compared to a more classical network based on simple correlations (i.e., “relevance network” [18]). According to the result of a node clustering combined with biological validation, the structure of the network based on partial correlations was found to be more consistent to prior biological knowledge than the one based on simple correlations (see section “Materials and Methods” for further details on this comparison). This can be explained by the fact that partial correlations focus on direct correlations only, discarding indirect links due to a common strong correlation with a third gene.

A bootstrap approach was used to estimate partial correlations. In a previous simulation study (not shown), the robustness of this approach was assessed: simulated data were generated with a given correlation design corresponding to a GGM. The estimation of the partial correlations from the bootstrap approach was compared to the real model, and about thirty observations were needed to obtain stable and accurate estimations. Thereby fifty-six observations were considered as a consistent dataset and the resulting network was indeed reliable. Finally, once the partial correlations were estimated, a Bayesian significance test was performed to discard non-significant links, i.e., edges that correspond to partial correlations, which are too small, as described in [8].

The obtained network contained 272 nodes (the genes) and 4,690 edges between significantly co-expressed genes. The network density that corresponds to the number of edges, divided by the number of node pairs was equal to 6.4%. The network was completely connected; any node in the network could be reached from any other node by a path passing along the edges.

### Important Nodes

**Figure 2:**
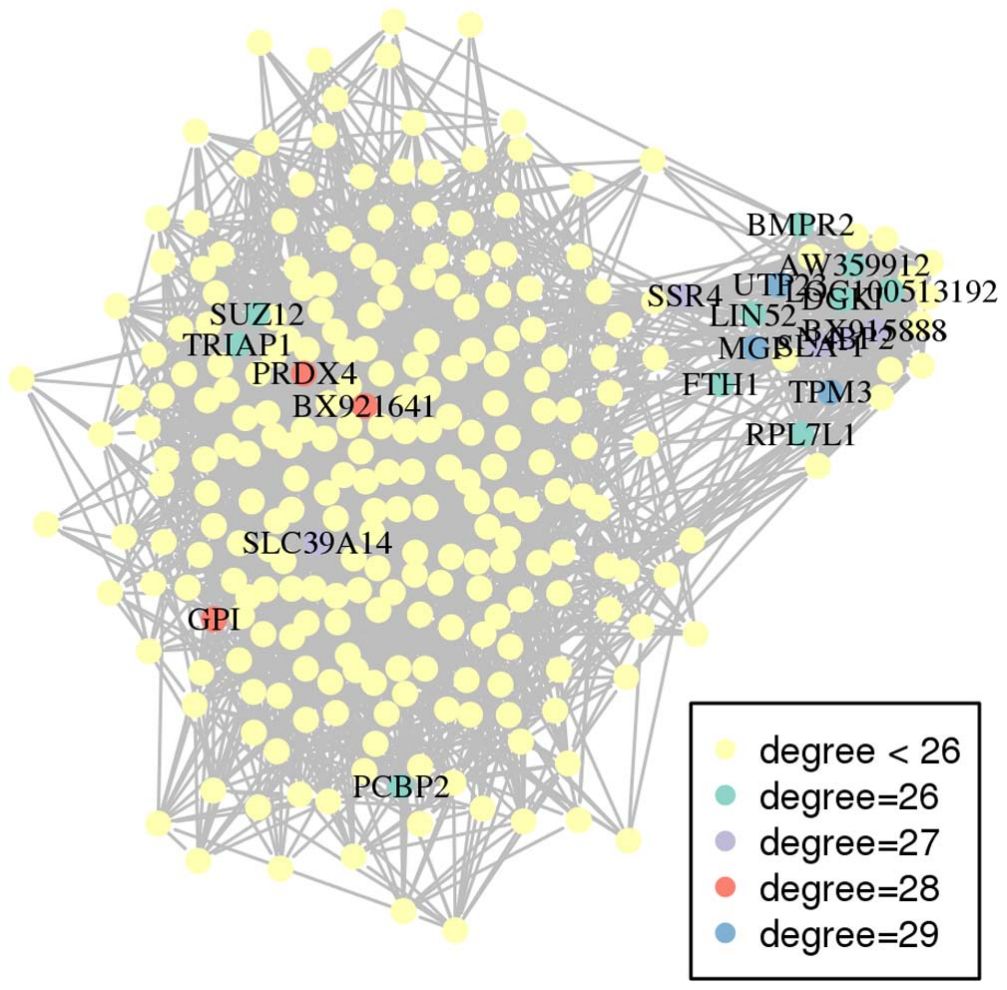
The co-expression network where hubs are highlighted. The names are also given. The list of hubs is available in Table S1.

**Figure 3:**
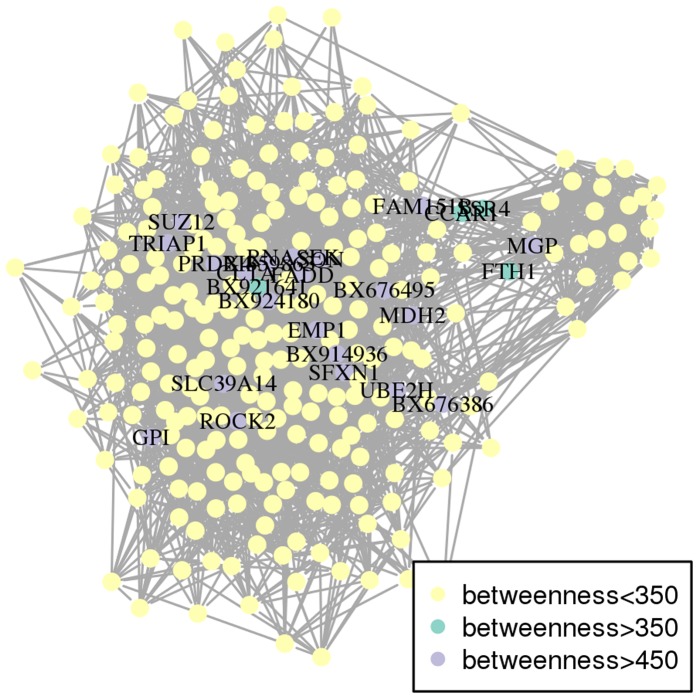
The co-expression network where genes with high betweenness are highlighted. The names are also given. The list of genes with high betweenness is available in Table S1.

**Figure 4:**
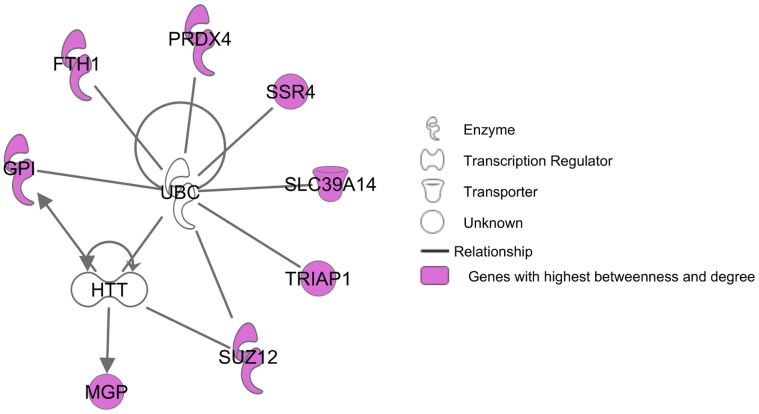
Bibliographic network obtained with the 8 annotated genes out of the 9 having the highest degree and betweenness. This network (score 68 : this score is a quality score given by IPA; see “Biological validation” in section “Materials and Methods” for further details about this score ) is related to regulation between several signaling pathways, metabolism and cell cycle apoptosis.

The network properties are useful for highlighting some key nodes/genes. “Hubs” are often viewed as important nodes in a network: they are nodes with the largest degrees, i.e., nodes that share the largest number of connections with the other nodes. The network contained 21 hubs having a degree larger than 26; three of them had a degree equal to 29, three to 28, five to 27 and ten to 26. Additionally, the node betweenness was also calculated: it is the number of shortest paths between two nodes that pass through the node under examination. Hence, twenty-five nodes with a high betweenness (here greater than 350) were those, which connect the network: if removed, the network is more likely to be disconnected. Finally, nine genes were found to be both hubs and nodes with a high betweenness. Among these nine genes, eight were annotated and found to be connected together by the ubiquitin and the huntingtin proteins: they might correspond to genes with a connecting role between metabolic and/or signaling pathways (see Section “Discussion” for further details). Hubs and high betweenness genes are listed in Table S1 and are emphasized on the network in Figures 2 and 3. Figure 2 shows that the densest part of the network contained most of the hubs (14/21) and conversely, half of its genes (14/28) are hubs. Figure 3 emphasizes the twenty-five genes that had a high betweenness. Hubs and genes with high betweenness did not provide enrichment for any given molecular function. On the contrary, the hubs have various functions such as growth factor, enzyme, transporter, component of the cytoskeleton…. Nevertheless, nine genes were hubs with a high betweenness, out of which eight were annotated. Biological enrichment was tested with IPA software for these genes, which are important for connecting the other genes together. One bibliographic network was obtained, including twenty-five out of the twenty-seven genes (score 68 : this score is a quality score given by IPA; see “Biological validation” in section “Materials and Methods” for further details about this score ) involved in the regulation between several signaling pathways, metabolism and cell cycle/apoptosis. This network is given in Figure 4.

### Network Clustering

Node clustering was performed using several approaches: modularity optimization, kernel 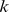-means and kernel SOM (see Section “Materials and Methods” for further details and references on these methods). The obtained gene clusters were systematically tested for their enrichment of Gene Ontology categories with WebGestalt. This first step lead to select the network based on partial correlations instead of simple correlations and the clustering based on modularity optimization. The clustering obtained from the modularity optimization [19] was the most consistent with biological knowledge, and was thus the one retained for further analysis. It was also the one with the highest modularity, equal to 0.4.

**Figure 5:**
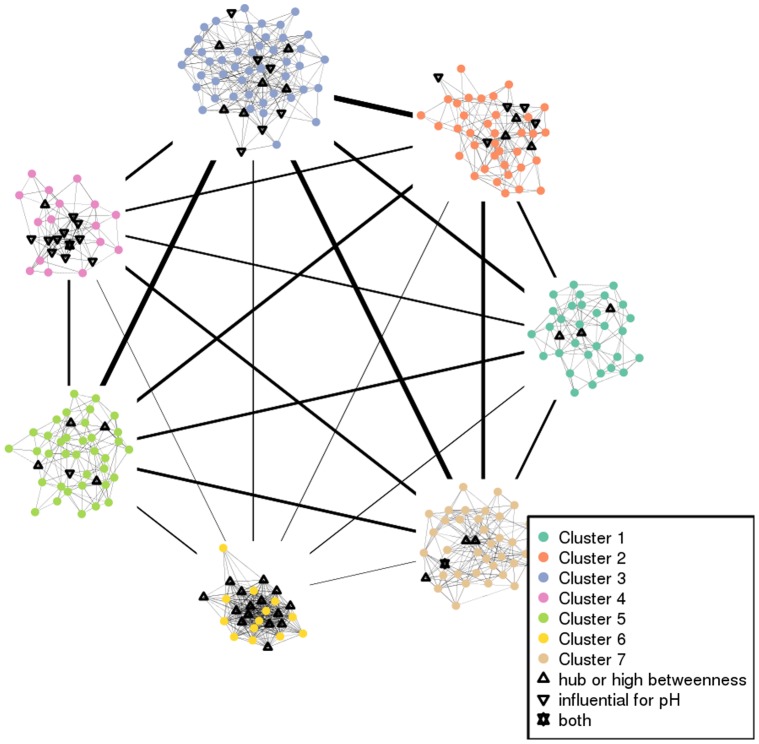
Simplified representation of the network. Special nodes are highlighted according to their level of degree or betweenness, and/or their partial correlation to a phenotype related to meat quality (pH 24 h after slaughtering). The line width between clusters is proportional to the number of links between the nodes of the corresponding clusters.

Seven clusters were identified that contained from 28 to 58 genes. Figure 5 provides a simplified representation of the network divided into the seven clusters.

Most hubs (14/21) belonged to cluster 6, contrary to the genes with a high betweenness that were almost equally allocated between the seven clusters. Only cluster 3 contained a larger number of genes with a high betweenness (six while the other clusters contained two to four genes with a high betweenness). The biological relevance of each cluster, as a subset of genes, was first explored in terms of Gene Ontology as explained before. Only 45% of the 272 genes had an ontological annotation, so the biological relevance was verified using Ingenuity Pathways Analysis (IPA) to construct bibliographic networks. Up to 67% of the 272 genes were eligible for network analysis by IPA. The correspondence between the clusters and the networks from IPA is given in Table 1. The relevance of the list of genes for all clusters was high, with about 83% of the eligible genes belonging to a single IPA network (at least 71%, and up to 94%). This means that the sets of genes obtained by an automatic clustering of the co-expression network have a strong consistency with the literature: they are most probably reliable for inferring the biological function of yet unknown genes according to the cluster to which these genes belong.

**Table 1:** Correspondence between clusters found by node clustering and bibliographic network.

Cluster	Nb of genes inthe cluster	Nb of genescalled eligible	% of the eligible genesinvolved in the samebiological network	Score	Main biological functions associated with the network
1	33	24	71	49	Development, cell death
2	44	28	93	70	Folding of protein, neuromuscular disease
3	58	38	71	65	Stress response, muscle development protein synthesis
4	28	17	94	44	Cell cycle and cell death
5	41	30	80	61	Gene expression cellular maintenance
6	28	19	84	40	Muscle and connective tissue development regulation of RNA expression
7	40	26	88	59	Cell death
Total	272	182 (67%)	mean is equal to 83%		

The list of genes for each cluster was submitted to IPA software and only one biological network was obtained. The eligible genes are those with a gene name accepted by IPA for having biological functions. An average of 83% of the eligible genes were included in the same network. IPA gives also the top biological functions associated with each cluster.

### Relations between the Co-expression Network and a Phenotype of Interest

In order to assess if a correlation existed between the network topology (the clusters) and a phenotype of interest (muscle pH), the partial correlations between pH and gene expressions were calculated. The pH values of muscle tissue after slaughtering are related to meat quality. Only the ultimate pH value (measured 24 h after slaughtering) is available but it is known to be not accurate enough to discriminate the metabolic processes underlying the way pH declines [20]. The purpose of the present section is thus to understand the relation between our set of genes (that are under eQTL control), their functions and this phenotype.

First, a Moran’s permutation test was performed to assess the correlation between the network structure and the partial correlation values. This test aims at answering the following question: “Do nodes that are linked in the co-expression network have a tendency to be similarly correlated with pH? ” To that aim, Moran’s 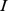 [21] was calculated: Moran’s 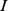 is a weighted correlation coefficient used to detect departures from spatial randomness. A statistical test, based on random permutations, as described in [22], was performed to assess the significance of its value: it was proven that Moran’s 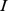 was significantly larger in our network than if the partial correlations were distributed among the nodes independently from the network structure. This means that nodes linked in the network have similar correlations with pH.

**Figure 6:**
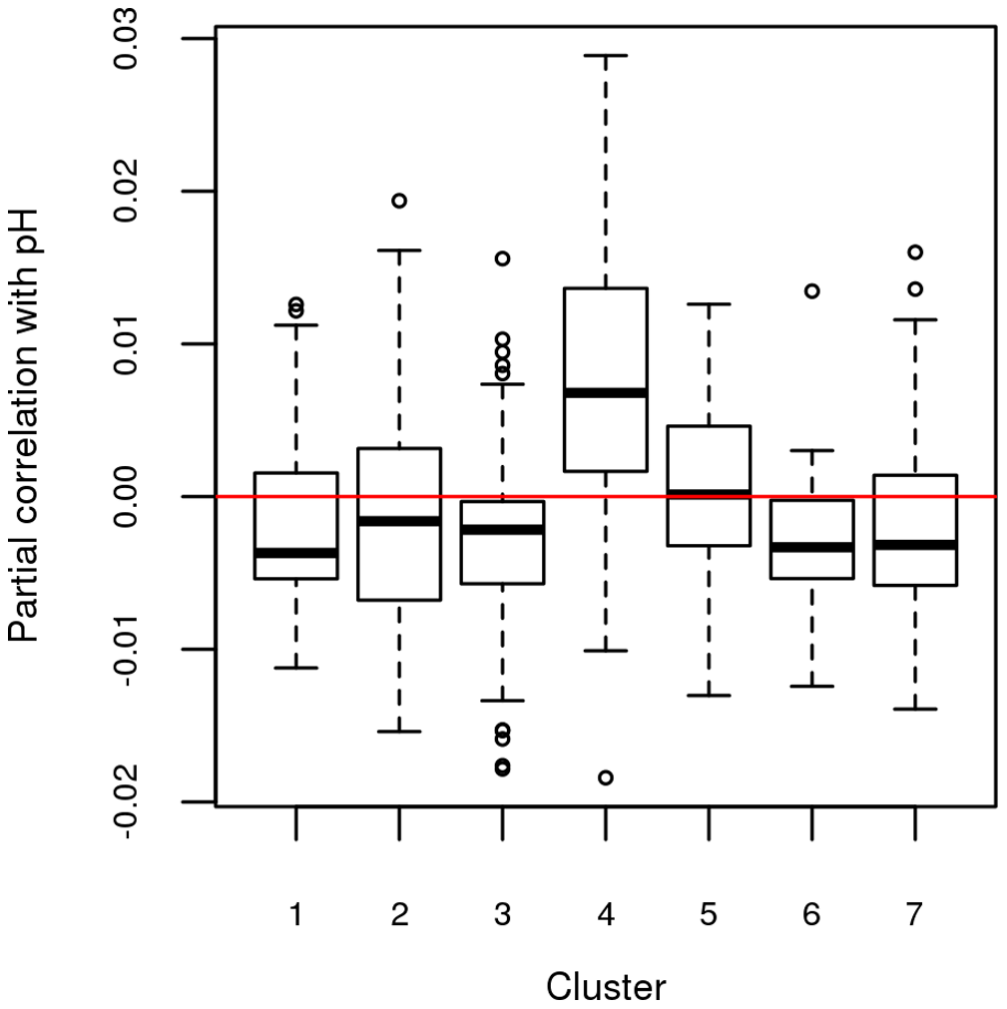
Boxplots of the partial correlations between the gene expressions and the pH for each cluster. Cluster 4 is significantly correlated with the pH phenotype (p-value is equal to 0.001).

Moving down to the cluster level, it was then possible to show that genes in cluster 4 had a significantly higher partial correlation with the pH than the genes in the other clusters (Figure 6), according to a t-test. Note that the values of the partial correlation should not be compared to the values of the correlations: a strong correlation between two genes results in a correlation coefficient close to one or to minus one but a similar behavior is not to be expected from the partial correlations: these quantities are conditional correlations and are thus much smaller than the direct correlations. To check that the other clusters had no correlation with the pH, the absolute values of the partial correlation with the genes expressions in cluster 4 were also calculated. This confirmed that cluster 4 is significantly more connected to the variation of muscle pH than the genes in the other clusters, according to a t-test. With a bibliographic network IPA analysis, cluster 4 was found to be related to cell death and cell cycle, with three genes (*GPI*, *B2M* and *XIAP*) essentially regulating cell death. Further discussion is provided in Section “Discussion”.

**Figure 7:**
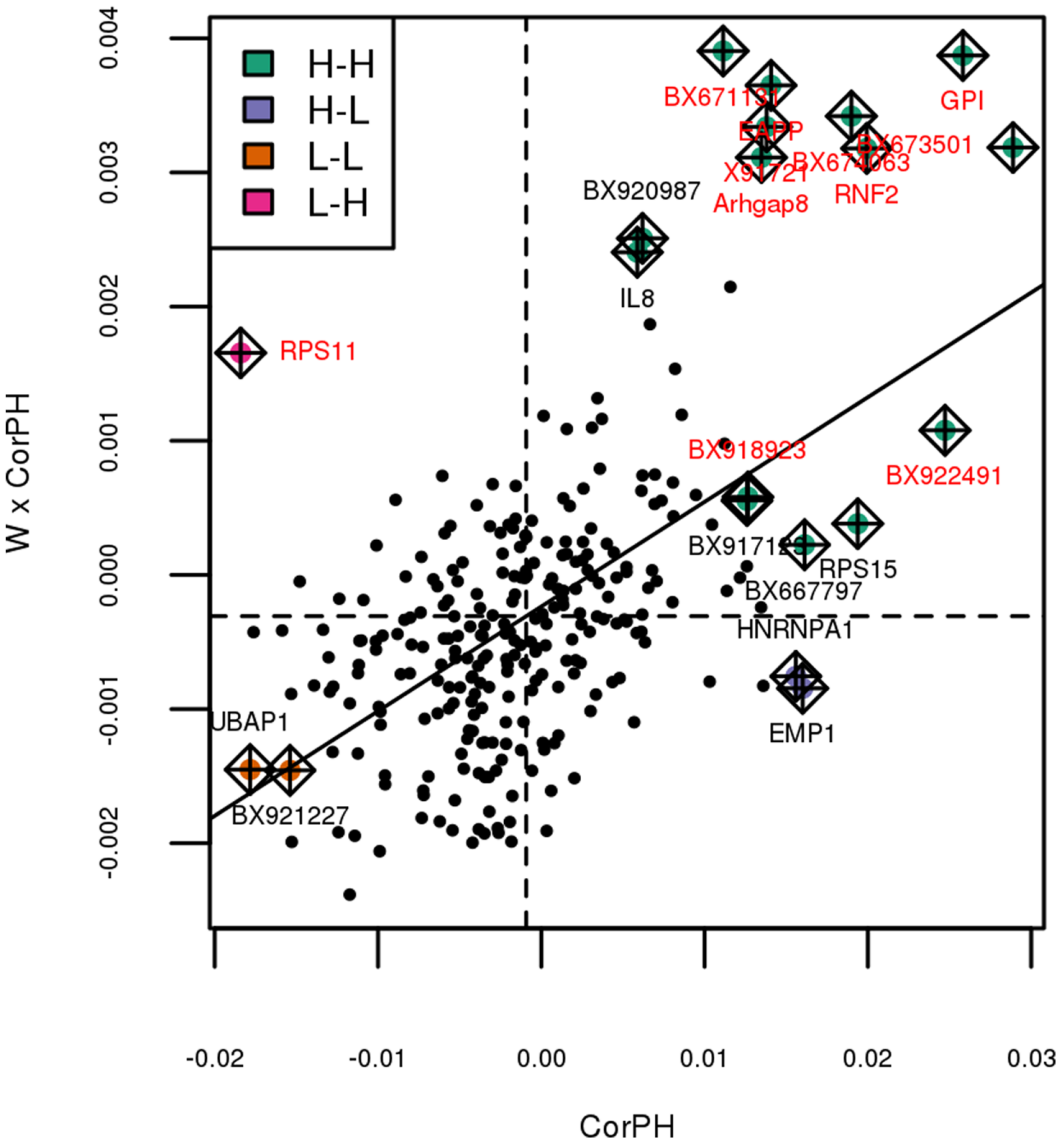
Moran’s plot of the partial correlation between pH and expression levels in the co-expression network. Influential nodes are displayed in color and their names are given. Influential genes labeled “H–H” have a strong positive correlation with pH (above the mean) and are linked to genes having a strong positive correlation with pH (above the mean); influential genes labeled “H–L” have a strong positive correlation with pH (above the mean) and are linked to genes having a strong negative correlation with pH (below the mean); influential genes labeled “L–H” have a strong negative correlation with pH (below the mean) and are linked to genes having a strong positive correlation with pH (above the mean); influential genes labeled “L–L” have a strong negative correlation with pH (below the mean) and are linked to genes having a strong negative correlation with pH (below the mean). Genes in red are in cluster 4, the cluster that is the most correlated to pH.

**Figure 8:**
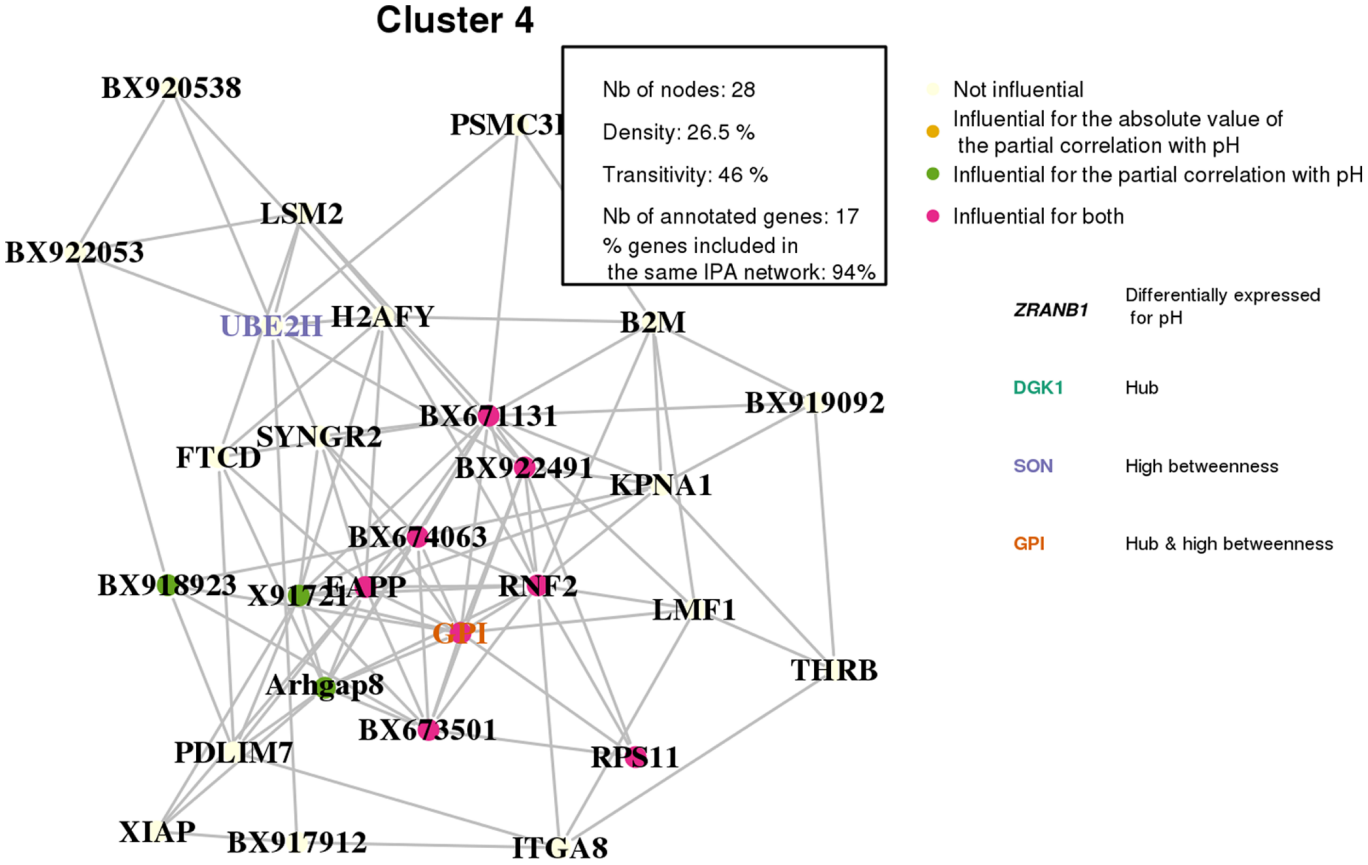
Detailed display of cluster 4. Nodes that are influential for the partial correlation with pH, as well as nodes that are important for the structure of the graph (hubs, high betweenness), are highlighted. The other clusters are displayed similarly in Supplemental Material, Figures S1, S2, S3, S4, S5, and S6.

**Figure 9:**
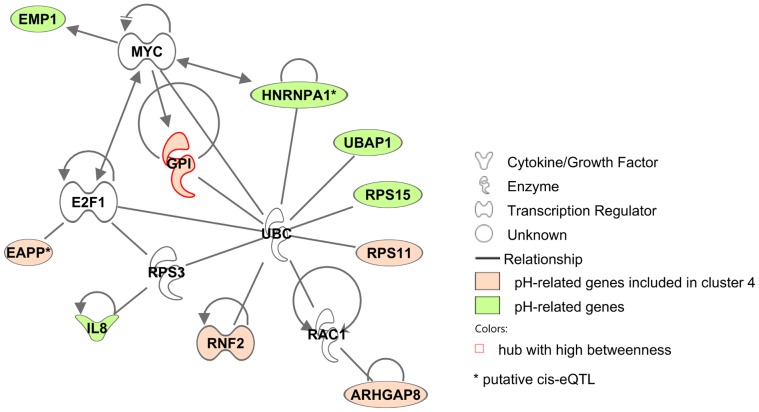
Bibliographic network obtained with 10 pH-related genes. Pink nodes are the genes included in cluster 4; the other nodes are green. Finally, white nodes are the genes included by IPA to define the network but not shown to be regulated by an eQTL in our previous study.

Finally, the gene level was also studied by using Moran’s plot to detect influential genes [23]. Moran’s plot displays the average values for partial correlation with pH in the neighborhood of a node as a function of the partial correlation with pH for this node (Figure 7). In this plot, the way a gene is linked to pH is analyzed together with its neighboring genes in the network. For instance, *GPI* is an influential gene in the quadrant “H–H”: this means that, not only is its expression highly correlated to the pH value but its neighboring genes also have an expression that is highly correlated to the pH value. Indeed *GPI* has an expected function (glycolysis) involved in the regulation of pH. A more complete discussion about *GPI* is provided in Section “Discussion”. Thereby, influential nodes for pH [24] were extracted from Moran’s plot; most of them belonged to cluster 4 (Figure 8). Twenty genes were detected as influential in Moran’s plot and eleven of them were in cluster 4 (out of twenty-eight genes classified in cluster 4). From these twenty genes, ten genes were eligible by IPA and were all included in the same biological network (Figure 9).

Table S1 contains the gene description (accession number, gene name, gene description, heritability, number of eQTL, putative cis-eQTL, genomic localization), along with the results of our analysis (degree, hub, betweenness, cluster, differentially expressed for pH, influent for partial correlation with pH, influent for absolute value of partial correlation with pH).

## Discussion

The overall methodology described in this paper is a pipeline of statistical methods to gain knowledge from raw data on a selected (here genetically regulated by eQTL ) set of genes. This pipeline includes three steps.

- The definition of a co-expression network to give a simplified and significant view of the interaction structure between those genes. This network can be used to identify key genes.
- A clustering of the nodes based on this network, built only from significant relations between genes. It helps to identify relevant groups of genes with a common function.
- External information, related to a trait, has been integrated into this network. In our case, the network structure was proven to be correlated with the value of the correlation between the gene expression and the pH. Moreover, the correlation between gene expression and pH, used together with the network structure, helped to identify important genes related to pH. Most of the genes that were identified as related to the pH, were also involved in a same cluster with other genes sharing biological functions (cluster 4, see Figure 8). Moreover, all the annotated genes influential for their partial correlation with the pH were also involved in one biological network (Figure 9).

### A Relevant Strategy to Model a Gene Network

Inferring a co-expression network directly from gene expression data can be achieved with a large number of statistical approaches: among them, the most studied are probably Gaussian Graphical Model (GGM) [25], Bayesian networks [26], [27] or mutual information networks [28]. As network inference is a topic of much interest, several packages have also been developed for the free statistical software R : for example, **GeneNet** [8] is a Graphical Gaussian method including a Bayesian significance test; **GGMselect** is a sparse Graphical Gaussian approach (see Baraud et al. 2009: http://fr.arxiv.org/abs/0907.0619); **minet** [28] is an R/bioconductor package using mutual information; **bnlearn** [29] is based on Bayesian network learning.

In the present article, a GGM was used, as implemented in the R package **GeneNet** to infer the network from a bootstrap approach and a Bayesian test. GGM is based on the estimation of partial correlations. As mentioned in the review of [30], the use of partial correlations instead of simple correlations is more appropriate to measure the dependence between variables. The correlation has to be preferred when the independence between variables is the targeted problem. Hence, the method combines the availability of a dedicated R package, with good performances, compared to several other alternatives [31].

### Extracting Putative Key Genes from the Network

The analysis of the network had two main purposes: first was to highlight key genes for co-expression, and second was to gain knowledge on unknown genes. Key genes were found by a direct analysis of the structure of the network, or by superimposing information (related to a phenotype of interest) to the network.

Several characteristics related to the network structure can be calculated to provide insights about key genes [9]. For instance, hubs are genes with the highest degree and have been proven to organize the proteome by connecting biological processes [10] or to be implicated in cancer [32]. The betweenness centrality measure [33] is well known in social network analysis but less standard in biological network analysis. Betweenness is an interesting criterion as nodes with a high betweenness form a strong network connection and hence have a strong impact on the network structure. Therefore the modification of these genes might have a large impact on underlying biological functions. This fact has already been described in medicine [11], in a study on network evolution [34], and in protein-protein interaction networks [35].

A few examples of extracted genes are provided thereafter. Their possible relevance in the way a muscle functions, or their possible involvement in pH values, is emphasized when existing studies have previously described that point. These examples aim at illustrating that some genes, which were automatically extracted thanks to the co-expression network model, showed a strong relevance for the understanding of the considered biological process. In our study, nine genes were both hubs and nodes with a high betweenness (*TRIAP1*, *SUZ12*, *PRDX4*, *GPI*, *SSR4*, *FTH1*, *MGP*, *SLC39A14* and *BX921641*). The eight that were annotated, were connected together by the ubiquitin and the huntingtin proteins (see Figure 4). A hypothesis is that these nodes could correspond to genes with a connecting role between metabolic and/or signaling pathways. These two proteins (ubiquitin and huntingtin) are ubiquitous and involved in several pathways. As explained by [36] in a review dedicated to the function of the huntingtin protein, huntingtin may interfere with transcriptional mechanisms common to many genes including markers of terminal muscle differentiation, metabolic enzymes (as *GPI* in cluster 4), signal transduction molecules, and fast myofibrillar fibers (as troponin 1 present in the cluster 2). Some mRNAs (e.g., ubiquitin-conjugating enzymes) concurrently increased in muscle, implying a cellular stress response.

Globally, extracted genes were either:

- annotated genes known to be involved in muscle physiology or even in meat quality. For example, *GPI* (glucose-6-phosphate isomerase) was a hub, a gene with a high betweenness, and an influential node for the correlation with pH. *GPI* protein is known to be involved in energy pathways, glycolysis and gluconeogenesis and these pathways are well known to be related to meat quality. Moreover *GPI* is localized on chromosome 6 at the position of several QTLs (Quantitative Trait Locus) affecting ultimate pH in loin muscle. Hence proposing *GPI* as a positional and functional candidate gene makes sense [37].
- annotated genes never cited for being involved in muscle physiology and even less in meat quality. For example, *MGP* (Matrix gla protein) was the hub with the highest degree and also had a high betweenness. [38] proved that it is involved in the inhibition of the switch from vascular smooth muscle cell in osteoblast-like cells and also calcification of arteries. To our knowledge, nothing has been described for *MGP* in skeletal muscle except in our first study [15] in which we identified a putative *cis*-eQTL for this gene.

From the relevance of the previous conclusions, it seems therefore interesting to focus on:

- genes which are un-annotated and whose function is therefore unknown. For instance, *BX921641* is a hub and also the gene with the highest betweenness. In further studies, it would be interesting to investigate the function of this gene in muscle tissue.

The main biological finding of this study, compared to a bibliographic gene network study (like IPA), lies in the fact that the combination of statistical methods is able to be used in the same analysis for all the genes of interest, either functionally known or not. Among the 56 genes highlighted as being “important” (hubs, high betweenness, or high influence for their correlation with a trait), only 67% are functionally known, and the others would have been discarded with solely a standard analysis, based on bibliographic knowledge.

### Gaining Knowledge from the Gene Clustering

The second step of the proposed pipeline was to elucidate the biological meaning of the gene network. The complete network with 272 genes was difficult to read except for the densest part of the network, as it is usual for networks with more than a hundred nodes. Indeed, as explained in [39] the standard way to display networks, i.e., by the use of force directed placement algorithms such as the algorithm described in [40] is not enough to identify a structure inside the network. Indeed, groups of genes (also often called “modules”) that are the most densely connected (and comparatively less connected to the other nodes) can often not be identified visually. The general structure of the network, decomposed into sub-graphs, can be revealed using node clustering. [41] provided a very complete review of methods used to cluster the nodes of a network and [42] compared several popular methods to cluster protein-protein interaction networks. This promising approach aims at revealing the biological structure behind the statistical one: it is a well-known fact that biological functions are carried out by modules in interaction networks [43]. Moreover, as pointed out by [17], network inference is more robust when dealing with modules than with individual interactions. Here, this approach was proven to be highly powerful to cluster together genes with common biological functions. Several methods to cluster genes were tested and biologically compared to each other with systematic measurements of ontological enrichment with the WebGestalt software [44] (see Section “Materials and Methods” for further details). The best clustering was also submitted to IPA. Nearly all the genes eligible to be submitted to IPA (about 80%) were included in a same bibliographic network: one cluster corresponded to one IPA bibliographic network (Table 1). It thus gave clues to the biological role of a group of genes, including the unknown genes.

Each cluster extracted from this analysis is fully described in Figures S1 (cluster 1), S2 (cluster 2), S3 (cluster 3), S4 (cluster 5), S5 (cluster 6) and S6 (cluster 7). The legend of all these figures is the same than the legend of Figure 8.

### Integrating Additional Information Related to a Phenotype of Interest

It is of major interest to add phenotypic information to a co-expression network in an integrative strategy to combine different levels of information. Moreover in our context, the 272 genes have been identified, so as to have their expression genetically regulated by eQTL, and moreover, without being selected to be differentially expressed according to a phenotype [15]. An important biological result of this work was to be able to merge co-expression information with the correlation to a trait of interest (muscle pH). In addition to the structure of the gene network, [1] showed that the relation with a phenotype or a trait of interest can be helpful to decipher the molecular interactions underlying a complex trait. When the phenotype is discrete, such as healthy/cancer, methods have been proposed such as COSINE [12]: differentially expressed genes (DEG) and differential correlation between groups are combined in this method. When the genes under study are not DEG, the relation between the selected genes and the phenotype may be weak, and such an approach is not usable anymore. In our study, this issue was addressed by using a labeled network, i.e., a network whose nodes are labeled by additional information, because this approach combines interactions between genes and correlations to a phenotype of interest, in the same model. It does not rely on individual tests for each gene and it is thus better suited for understanding the process in its totality and to extract groups of genes related to the phenotype.

Finally, the pH reflects the acid-base homeostasis of a living system (muscle tissue). From the 20 genes found to be important for the partial correlation with the pH, only *GPI* has an expected function (glycolysis) involved in the regulation of pH: accelerated postmortem glycolysis affects a rapid pH fall. Moreover, *GPI* gene is included in cluster 4, which has the highest correlation with the variation in pH. This gene seemed to be the most important gene in this cluster. *GPI* is altogether a hub with a high betweenness, and a gene highly correlated to pH variation. In the postmortem muscle of pigs, the energy metabolism shifts from an aerobic metabolism of lipids to anaerobic metabolism of muscle glycogen. Unfortunately, the way the ultimate pH decreases is rather difficult to control. Nevertheless, ultimate pH is often measured as a consequential factor [45]. With the identification of *GPI*, which was central in a network related to pH, geneticists are offered interesting proposals for further experiments. *GPI* gene is a functional and positional gene candidate to explain effects observed at a QTL position on chromosome 6 on muscle pH values [37]. The expression of *GPI* is genetically regulated by two *trans*-eQTL on chromosomes 5 and 8 in our context, and no *cis*-eQTL was identified on chromosome 6 [15]. With a bibliographic network from IPA analysis, cluster 4 was found to be related to cell death and cell cycle: this IPA network included 78% of the annotated genes classified in cluster 4. Intracellular pH has an important role in the maintenance of normal cell function, and cellular modifications leading to pH changes have been implicated in both cell proliferation and cell death [46]. In our study, three genes essentially regulate cell death, (*GPI*, *B2M* and *XIAP*) suggesting a relation between pH regulation, which is a metabolic process, and cell death, which is the cell biological consequence of the failure of metabolism.

### Conclusion

An adequate combination of statistical methods, namely network inference using partial correlation under a Graphical Gaussian model, followed by node clustering, can lead to a significant improvement of our biological knowledge in the underlying biological functions of a set of genes. This approach is particularly useful in the context partial bibliographic knowledge where only half of the genes a given genome are still unknown. Moreover, this approach allows one to link the structure of the network to a phenotype, and then to identify key genes.

## Materials and Methods

### Data Description: eQTL Data

56 half sib pigs were produced from an F2 cross between two production sire lines (France Hybrides SA, St. Jean de Braye, France). Procedures and facilities were approved by the French Veterinary Services. Longissimus dorsi muscle RNA was extracted as described by [47]. The normalized data were submitted to NCBI (GEO accession number GSE26924). The eQTL analysis identified 335 eQTLs affecting the expression of 272 transcripts with an average heritability of 0.45 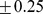 [15].

### Network Definition

In the Gaussian graphical model framework, gene expressions are modeled by a Gaussian variable 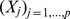, where 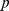 is the number of genes under study, with a covariance matrix 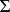. It can be proved that the partial correlations
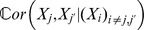
are obtained directly from 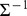 [25]. Many articles focus on the estimation of this inverse in the context of ill-posed problems: typically, the number of genes is much larger than the number of available observations, and directly inverting the empirical correlation matrix leads to numerical instability and bad estimations. One solution is the bootstrap estimation described in [8].

This approach was used, combined with the estimation functions implemented in the R package **GeneNet**. In this package, a shrinkage of the empirical covariance matrix 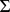 is performed prior to its inversion in order to limit numerical instability. This method simply consists in adding a small positive number to the diagonal of 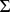. A bootstrap approach was then performed to obtain more robust estimates and made it possible to construct a co-expression network with the 272 genes. 4,000 bootstrap samples (size 20) were enough to obtain a stabilization of the estimation procedure. Then, the Bayesian test of significance, described in [8], and implemented in the R package **GeneNet**, was used to discard the smallest partial correlations. Finally, the network was displayed using the Fruchterman and Reingold algorithm [40] as implemented in the R package **igraph** [48].

### Network Clustering

Node clustering aims at finding densely connected groups of genes, called *clusters* or *modules*, in the network. As many methods to cluster the nodes in a network exist [41], three were chosen and compared. The first one consisted in optimizing the modularity: the modularity is a quality criterion for node clustering introduced by [19]. For a network with nodes 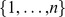 and edges weighted by 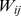 (where 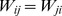 are either positive or null when there is no edge between 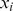 and 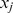) and for a partition 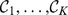 of the nodes, the modularity is equal to:
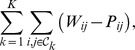
with 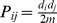, where 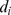 is the degree of 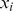 and 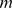 is the number of edges in the network. Its aim is to compare the actual weights of the edges to a null model where the edges depend only on the nodes degrees and not on their cluster. Hence, the higher the modularity, the more the edges are concentrated inside the clusters. In the case of unweighted networks (as in our study), 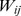 are either 1 (when there is an edge between 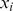 and 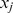) or 0. The modularity measure has already been used by [49] to recover functional modules in protein interaction networks with an optimization based on the original approach of [19]. Following the ideas of [50], the modularity was optimized by a simulated annealing algorithm, which is a more efficient approach for optimizing the criterion than the one proposed in the original article [19]. The annealing parameter of simulated annealing was chosen in an exponential search grid (varying from 10 to 105).

Modularity optimization was compared to alternative approaches that were based on *kernels* (see, among others, [51]) and all relate to spectral clustering [52]. More precisely, kernel 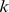-means and batch kernel SOM [53] were processed as implemented in the R package **yasomi** (development version available at https://r-forge.r-project.org/projects/yasomi).

To compare the three different methods (modularity optimization by simulated annealing, kernel 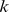-means and batch kernel SOM), the following methodology was used:

- for each of the three methods, several parameters were used to provide different results: the number of (initial) clusters of the algorithm varied from 4 to 12 (or, for kernel SOM, the data were projected on a 2-dimensional grid whose dimension varied from 2 to 4) and the heat kernel [54] and the Commute Time kernel [55] were tested;
- for each of the three methods, only one of these results was selected: the selection was made according to the modularity value (hence the modularity was also used as a quality measure for selecting the “best” clustering among the cluterings obtained with different tuning parameters);
- the resulting three clustering were subjected to a biological validation (as described in the next section).

Also, the same methods were also used to cluster a network based on simple correlations (“relevance network”, see [56]) in order to assess the relevance of the use of partial correlations compared to simple correlations.

Finally, as explained in the next section, biological validation leads to select the clustering based on the partial correlation network and on modularity optimization by simulated annealing.

### Biological Validation

In a first step, the WebGestalt software [44] provided a statistical enrichment analysis of the Gene Ontology Terms. The results were illustrated with an acyclic network of the ontology terms. Biological information given by GO enrichment is only based on a low number of genes (47%). However WebGestalt produced results faster than IPA and was more useful for the comparisons between different networks (defined from partial correlation or direct correlation) and different clustering algorithms with various parameters. A systematic comparison was performed to assess the biological relevance of the clusters obtained from these different methods: only the most relevant clustering was then analyzed, i.e., that which was obtained from the modularity optimization of the network built with partial correlations. A unique network (the one based on partial correlations) and a unique clustering (the one based on modularity optimization) was then kept, because they had the best agreement with biological knowledge, as computed by using the WebGestalt software.

In a second step, Ingenuity Pathways Analysis (IPA, https://analysis.ingenuity.com/pa) was used to explore and confirm the biological relevance of the identified clusters. IPA software contains a large bibliographic database with various types of links already identified between two genes (protein-protein interaction, ligand-receptor regulation, enzymatic modification, transcriptional expression regulation, etc.). IPA software was used to build biological networks, which correspond to the best possible arrangement of the eligible genes. 67% of the 272 genes (Table 1) are genes that have already been studied elsewhere and are annotated and referenced in IPA. For each IPA network a score is used to rank networks according to their degree of relevance to the “Network Eligible Molecules” (the input gene list) in the submitted dataset. The score is derived from a p-value (based on the hypergeometric distribution and calculated with the right-tailed Fisher’s exact test) and indicates the likelihood of the submitted genes to be found together in the same network due to random chance; for instance, a score of 2 indicates that there is 1 in 100 chance that the submitted genes are together in a network due to random chance.

### Using Spatial Statisics to Analyze the Link with a Phenotype of Interest

A final analysis focused on the relation between the network structure and a phenotype of interest (muscle pH). This analysis was performed by first calculating partial correlations between gene expressions and pH, using the same method that was described in the section “Network definition”. Then, tools coming from spatial statistics were used to extract influential genes. This approach is the one described in [22]. Briefly, it consisted first in calculating the Moran’s 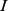 statistics to measure the correlation between the network structure and the phenotype of interest and to perform a permutation test to assess its significance and, then in finding the genes that had the strongest effects in the correlation between the value of the variable for a given node and the average value of this variable for its neighbors.

Additionally, the significance of a higher correlation with pH in one particular cluster compared to the others, was assessed by means of a t-test with level 1%, testing the difference in average between the absolute value of the partial correlation with pH in the considered cluster and the absolute value of the partial correlation with pH in the other 6 clusters.

## Supporting Information

Figure S1 **Cluster 1.** 71% of the genes eligible by IPA in cluster 1 belong to a single bibliographic network involved in cellular development and cell death (Table 1). The *FADD*, *CLTA* and *SFXN1* genes have a high betweenness in the structure of the graph. They are involved in apoptosis and cellular development respectively (*FADD*), in the process of receptor-mediated endocytosis (*CLTA*), and in cation transport (*SFXN1*). None of these genes are influential for the partial correlation with pH, but one of them, *DYSFIP1* is one of the three genes of the 272 to be a DEG according to pH value, while the two other DEG are in cluster 2. No functional information is available for *DYSFIP1*. It has been identified as a DEG in a skeletal muscle transcriptome study in mice to be down-regulated when mice are fed with a high-iron diet [57]. The color and font meanings are given in Figure 8.
(TIFF)

Figure S2 **Cluster 2.** 93% of the genes eligible by IPA in cluster 2 belong to a single bibliographic network (IPA) involved in folding of protein and neuromuscular disease with ten genes (*B2M*, *IL8*, *LDHA*, *OCLN*, *PDLIM7*, *PLOD1*, *SLC6A3*, *SPARCL1*, *VANGL1*, and *ZRANB1*). The muscle pH trait seems to be also related to some of the genes of this cluster without overall correlation of the cluster with pH values. *TRIAP1* and *SUZ12* are two genes of great importance for this cluster. They are both involved in the apoptosis process which was identified as one of the main functions regulated by the eQTL in the original study [15]. Apoptosis is a cellular response to stress which is tightly regulated by the protein p53. This protein may play a role as a “guardian of metabolic balance” between glycolysis and mitochondrial respiration for energy production [58], both pathways affecting muscle pH values. p53 adapts the cellular proliferation rate to the metabolic state. In the present study, p53 (*TP53*) gene expression was not identified to be genetically regulated but *TRIAP1* (TP53 regulated inhibitor of apoptosis 1 or p53-inducible cell-survival factor) plays an important role in response to p53 and determine cellular survival or death [59]. The other important gene in this cluster is *SUZ12*. In mice, the *SUZ12* gene was identified as being required for cellular proliferation and for EZH2 histone methyltransferase activity [60]. *SUZ12* is essential for the transmission of epigenetic marks [61], important to regulate embryonic development as the muscle developmental regulator MYOD [62]. It is very interesting to observe here that the two most important genes in cluster 2 are involved in very different but very important processes to regulate cell survival (*TRIAP1*) and regulation of muscle development (*SUZ12*). This supports the idea that the important genes may regulate complementary biological processes, even if biologists may be surprised to observe in he same cluster a direct link between the two genes. The color and font meanings are given in Figure 8.
(TIFF)

Figure S3 **Cluster 3.** This cluster is the biggest one with 58 genes but possesses the lowest density. It contains six genes with a high betweenness and two of them are hubs, but only two are known (*PRDX4* and *SON*). Sulfiredoxin (as PRDX4) is a new oxidative stress-induced antioxidant protein. Mechanistic studies further demonstrated that the integrity of the Srx Prx IV axis is required for sufficient activation and/or amplification of signaling cascades as MAPK pathways [63]. 71% of the genes eligible by IPA in cluster 3 belong to a single bibliographic network (IPA) involved stress response, muscle development and protein synthesis, in particular with seven annotated genes. Six genes, with five annotated (*HNRNPA1*, *UBAP1*, *CENPE*, *GNAI2*, and *THYN1*), have expression correlated with pH values. The color and font meanings are given in Figure 8.
(TIFF)

Figure S4 **Cluster 5.** 80% of the genes in the cluster are involved in cellular movement around *S100A4* and *CXCL12*. Four genes have a high betweenness, three are annotated: *MDH2*, *FAM151B* and *RNASEK*. *MDH2* (malate dehydrogenase 2, NAD, mitochondrial) gene plays a pivotal role in the malate-aspartate shuttle that operates in the metabolic coordination between cytosol and mitochondria. Moreover, *MDH2* is a putative cis-eQTL on chromosome 3. The same chromosomal location regulates another gene in this cluster, *ANXA7* (annexin A7). Both genes, *MDH2* and *ANXA7* are regulated by a miRNA, miR-135a. In skeletal muscle, miR-135a expression is modulated following ischemia [64]. The color and font meanings are given in Figure 8.
(TIFF)

Figure S5 **Cluster 6.** Cluster 6 is the cluster that can be visually identified as the densest part of the full network in the upper right part of Figure 1. Most hubs (14 genes) belong to this cluster. Four genes have a high betweenness and three of them are hubs and have a high betweenness: *SSR4*, *FTH1* and *MGP*. The specificity of this cluster is to be related to genes tightly transcriptionally regulated by the same complex of transcription factors. For example, *MGP*, *MYH2*, *BMPR2*, *STC1*, *FTH1* and *TPM3* expressions are regulated by NFAT (nuclear factor of activated T-cells). The gene transcription leads to protein synthesis, which is the biological function identified by IPA for this cluster (84% of the eligible genes are involved in protein synthesis and muscle development). The color and font meanings are given in Figure 8.
(TIFF)

Figure S6 **Cluster 7.** This cluster seems to be organized around *ROCK2* (Rho-associated coiled-coil forming kinase 2) and *PCPB2* (poly(rC) binding protein 2). *ROCK2* has high betweenness and was identified as being involved in cell death process (Shi and Wei, 2007). Cell death corresponds to the main biological function of this cluster identified by IPA, with 88% of the eligible genes. *PCPB2* is a hub in this cluster while *PCPB2* is also a cis-eQTL. This suggests a possible central role genetically controlled at the *PCPB2* locus itself. *PCPB2* with *AARS*, *PABPC1* and *SNW1* are involved in the gene expression regulation, especially via RNA spicing (Genecodis analysis, [65]). *SLC39A14* (solute carrier family 39 (zinc transporter), member 14) is both a hub and a high betweenness node. Zinc is an essential cofactor for hundreds of enzymes. It is involved in protein, nucleic acid, carbohydrate and lipid metabolism, as well as in the control of gene transcription, growth, development and differentiation. The Zn transporter SLC39A14 controls the G-protein coupled receptor (GPCR)-mediated signaling [66]. Cell signaling through GPCR (G protein-coupled receptors) plays a central role in mediating multiple signaling pathways. The color and font meanings are given in Figure 8.
(TIFF)

Table S1 **Description of the 272 genes.** Full gene description with accession number, gene name, gene description, heritability, number of eQTL, putative cis-eQTL, genomic localization, degree, hub, betweenness, cluster, differentially expressed for pH, influent for partial correlation with pH, influent for absolute value of partial correlation with pH.
(XLS)
