# Supplementary figures and images for: The Structure of a Gene Co-Expression Network Reveals Biological Functions Underlying eQTLs
Source: PLoS One. 2013 Apr 5;8(4):e60045. doi: 10.1371/journal.pone.0060045 (PMC3618335; doi:10.1371/journal.pone.0060045)

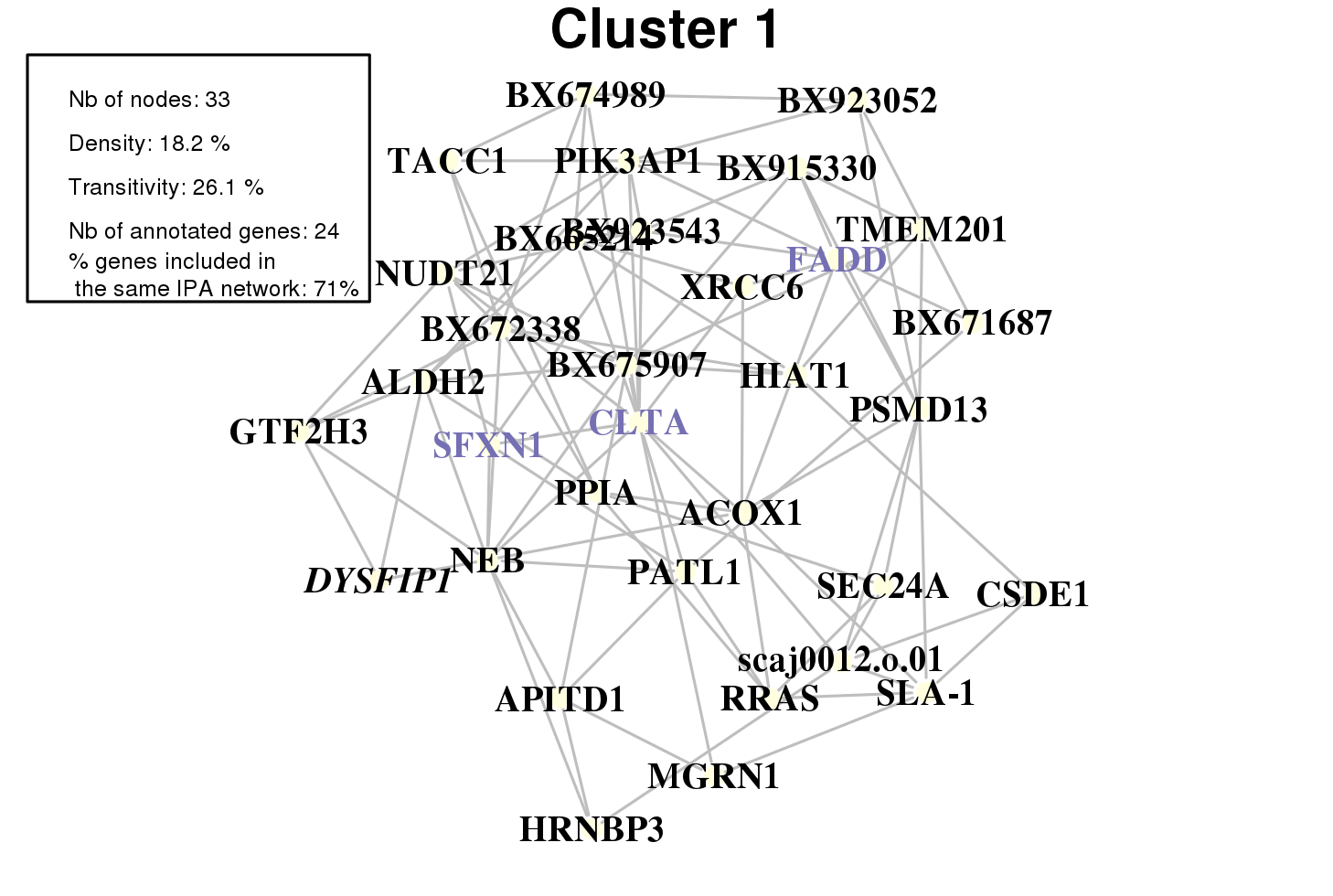

Supplement: Figure S1 — Cluster 1. 71% of the genes eligible by IPA in cluster 1 belong to a single bibliographic network involved in cellular development and cell death (Table 1). The FADD, CLTA and SFXN1 genes have a high betweenness in the structure of the graph. They are involved in apoptosis and cellular development respectively (FADD), in the process of receptor-mediated endocytosis (CLTA), and in cation transport (SFXN1). None of these genes are influential for the partial correlation with pH, but one of them, DYSFIP1 is one of the three genes of the 272 to be a DEG according to pH value, while the two other DEG are in cluster 2. No functional information is available for DYSFIP1. It has been identified as a DEG in a skeletal muscle transcriptome study in mice to be down-regulated when mice are fed with a high-iron diet [57]. The color and font meanings are given in Figure 8. (TIFF) [file pone.0060045.s001.tif]

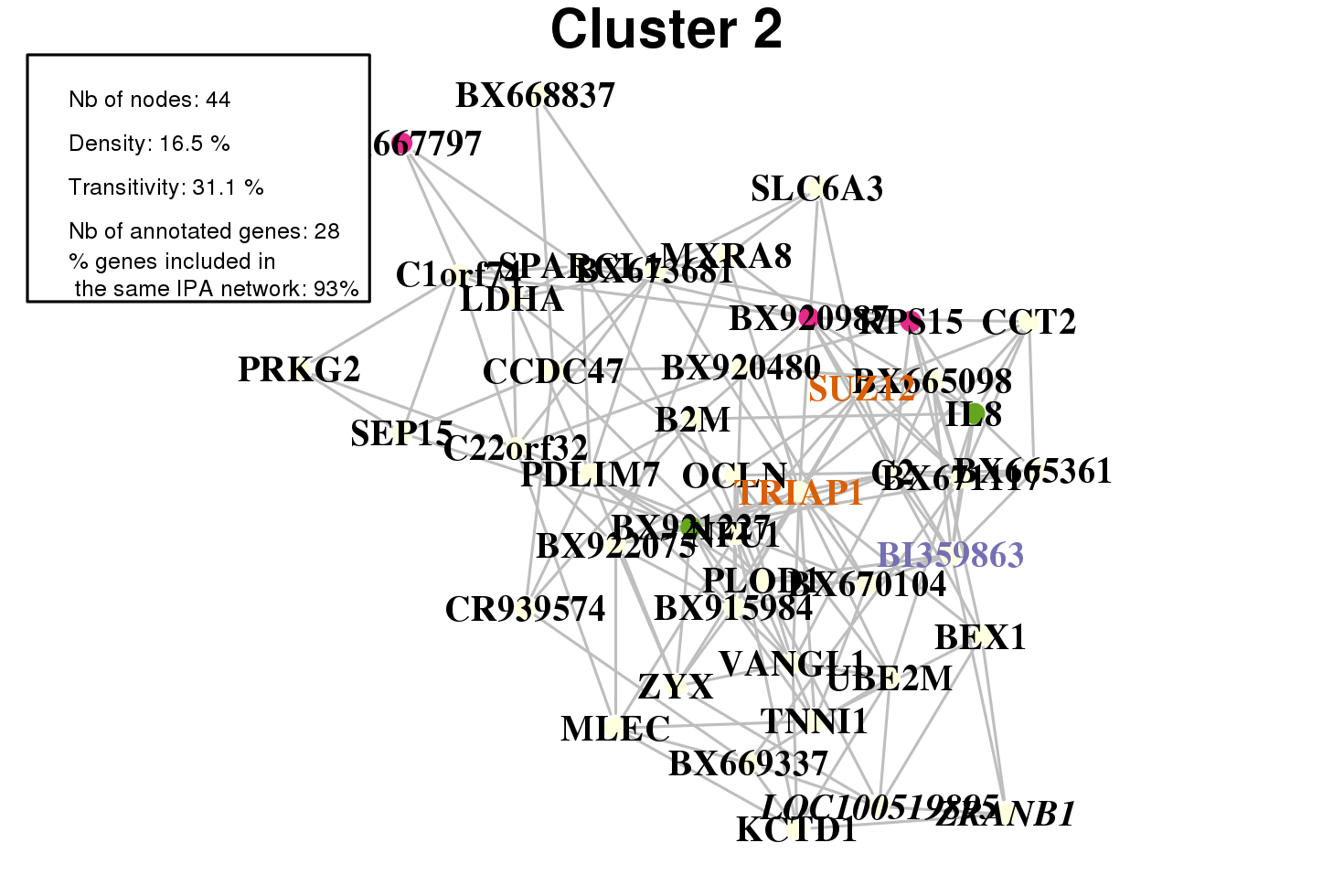

Supplement: Figure S2 — Cluster 2. 93% of the genes eligible by IPA in cluster 2 belong to a single bibliographic network (IPA) involved in folding of protein and neuromuscular disease with ten genes (B2M, IL8, LDHA, OCLN, PDLIM7, PLOD1, SLC6A3, SPARCL1, VANGL1, and ZRANB1). The muscle pH trait seems to be also related to some of the genes of this cluster without overall correlation of the cluster with pH values. TRIAP1 and SUZ12 are two genes of great importance for this cluster. They are both involved in the apoptosis process which was identified as one of the main functions regulated by the eQTL in the original study [15]. Apoptosis is a cellular response to stress which is tightly regulated by the protein p53. This protein may play a role as a “guardian of metabolic balance” between glycolysis and mitochondrial respiration for energy production [58], both pathways affecting muscle pH values. p53 adapts the cellular proliferation rate to the metabolic state. In the present study, p53 (TP53) gene expression was not identified to be genetically regulated but TRIAP1 (TP53 regulated inhibitor of apoptosis 1 or p53-inducible cell-survival factor) plays an important role in response to p53 and determine cellular survival or death [59]. The other important gene in this cluster is SUZ12. In mice, the SUZ12 gene was identified as being required for cellular proliferation and for EZH2 histone methyltransferase activity [60]. SUZ12 is essential for the transmission of epigenetic marks [61], important to regulate embryonic development as the muscle developmental regulator MYOD [62]. It is very interesting to observe here that the two most important genes in cluster 2 are involved in very different but very important processes to regulate cell survival (TRIAP1) and regulation of muscle development (SUZ12). This supports the idea that the important genes may regulate complementary biological processes, even if biologists may be surprised to observe in he same cluster a direct link between the two gen [file pone.0060045.s002.tif]

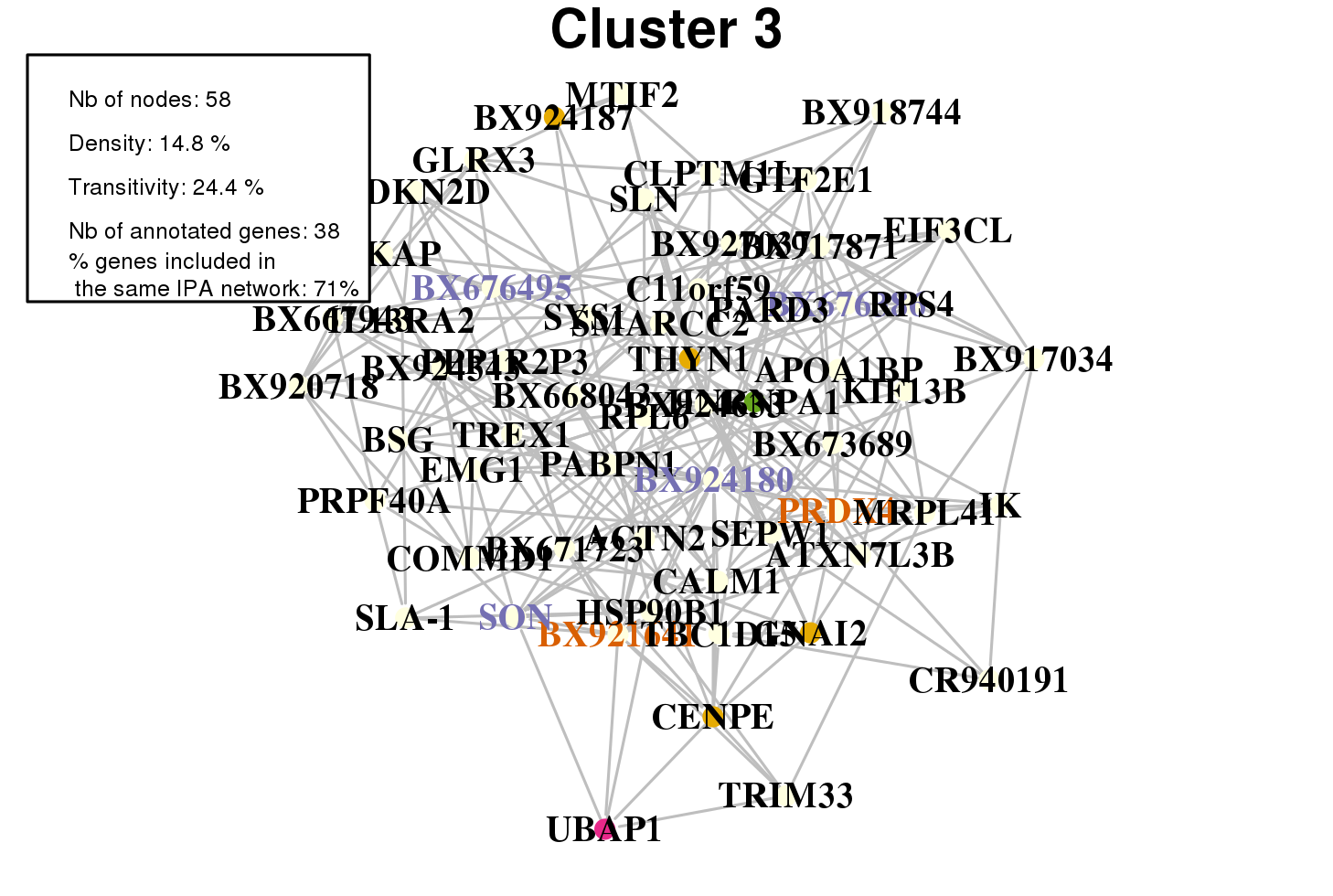

Supplement: Figure S3 — Cluster 3. This cluster is the biggest one with 58 genes but possesses the lowest density. It contains six genes with a high betweenness and two of them are hubs, but only two are known (PRDX4 and SON). Sulfiredoxin (as PRDX4) is a new oxidative stress-induced antioxidant protein. Mechanistic studies further demonstrated that the integrity of the Srx Prx IV axis is required for sufficient activation and/or amplification of signaling cascades as MAPK pathways [63]. 71% of the genes eligible by IPA in cluster 3 belong to a single bibliographic network (IPA) involved stress response, muscle development and protein synthesis, in particular with seven annotated genes. Six genes, with five annotated (HNRNPA1, UBAP1, CENPE, GNAI2, and THYN1), have expression correlated with pH values. The color and font meanings are given in Figure 8. (TIFF) [file pone.0060045.s003.tif]

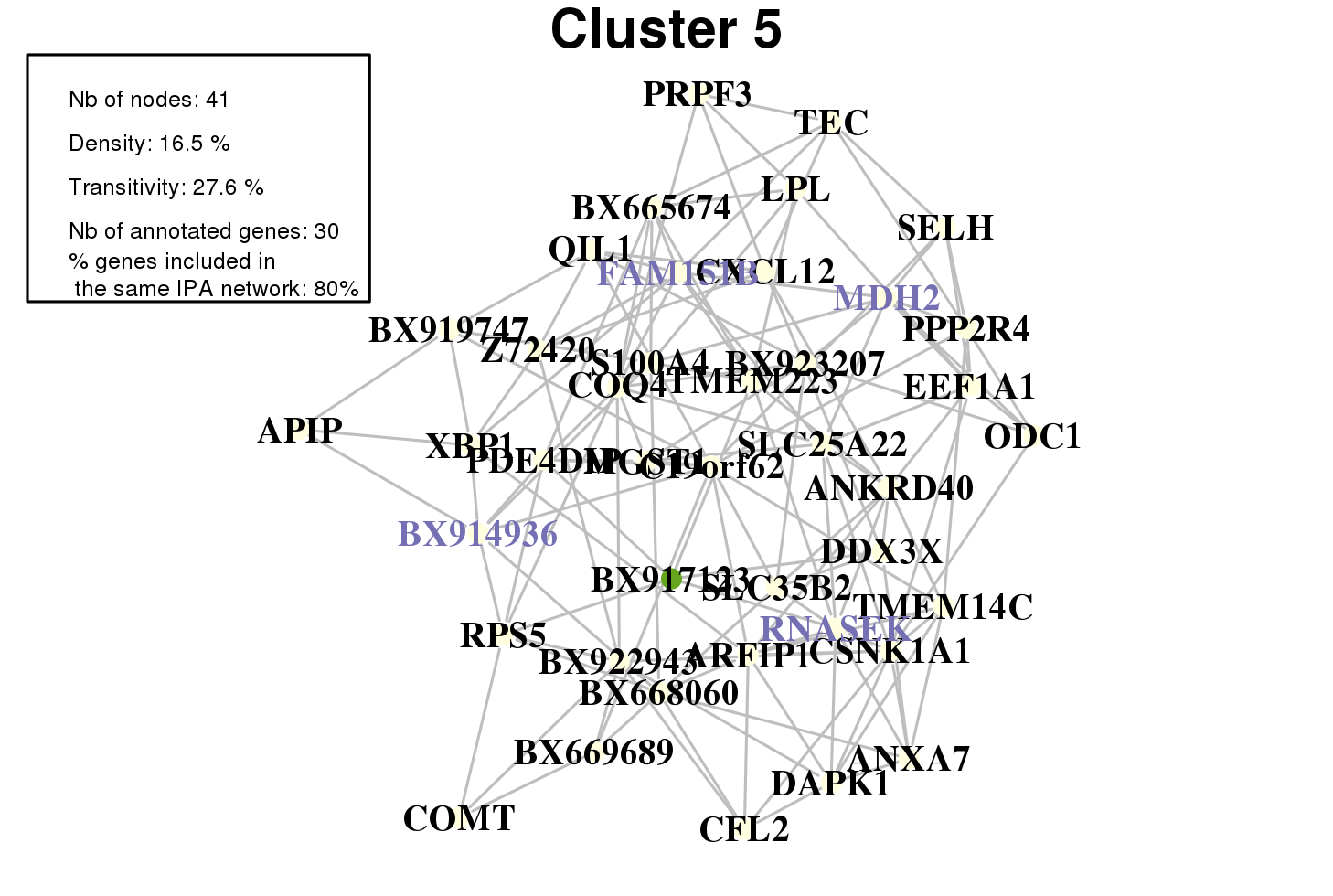

Supplement: Figure S4 — Cluster 5. 80% of the genes in the cluster are involved in cellular movement around S100A4 and CXCL12. Four genes have a high betweenness, three are annotated: MDH2, FAM151B and RNASEK. MDH2 (malate dehydrogenase 2, NAD, mitochondrial) gene plays a pivotal role in the malate-aspartate shuttle that operates in the metabolic coordination between cytosol and mitochondria. Moreover, MDH2 is a putative cis-eQTL on chromosome 3. The same chromosomal location regulates another gene in this cluster, ANXA7 (annexin A7). Both genes, MDH2 and ANXA7 are regulated by a miRNA, miR-135a. In skeletal muscle, miR-135a expression is modulated following ischemia [64]. The color and font meanings are given in Figure 8. (TIFF) [file pone.0060045.s004.tif]

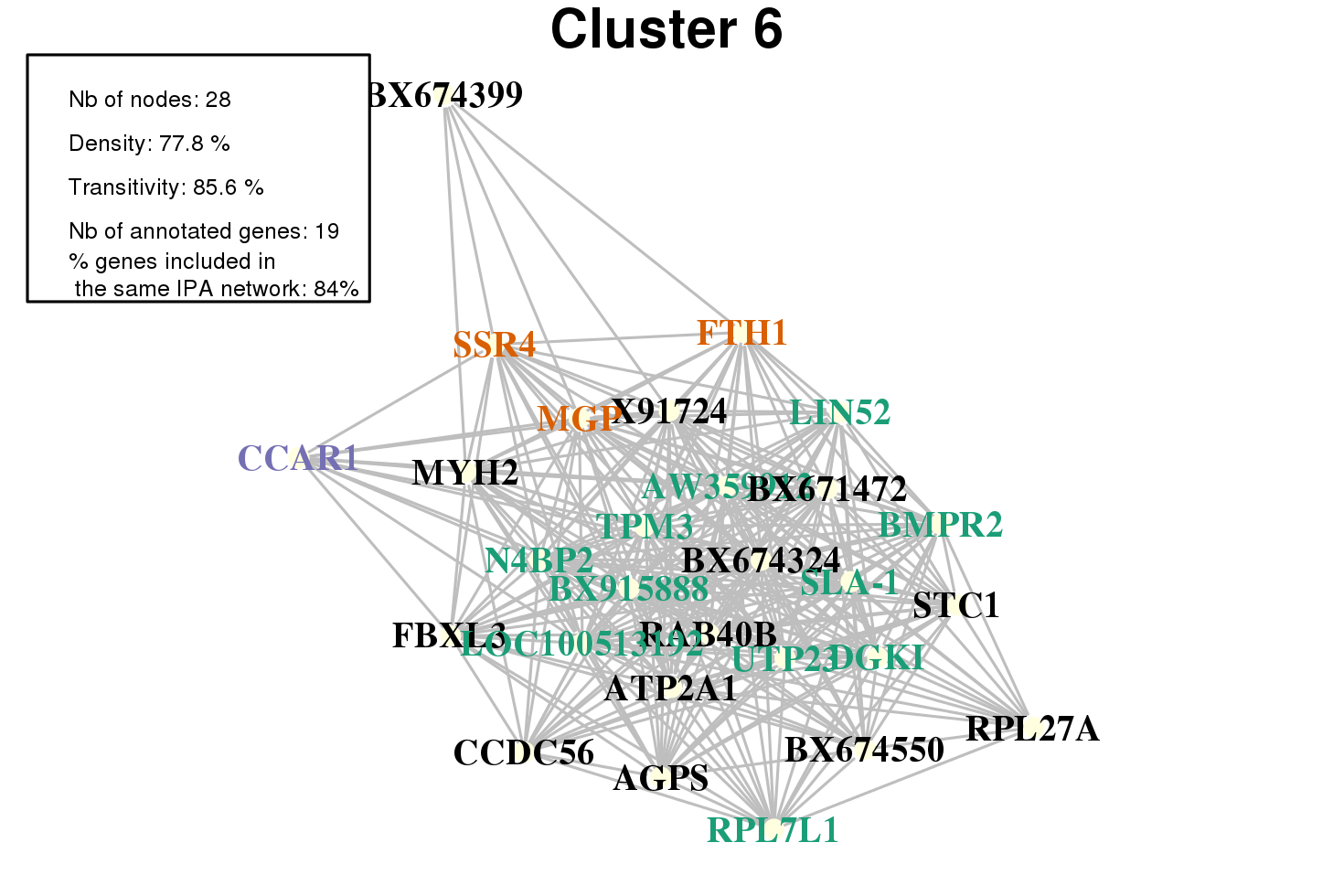

Supplement: Figure S5 — Cluster 6. Cluster 6 is the cluster that can be visually identified as the densest part of the full network in the upper right part of Figure 1. Most hubs (14 genes) belong to this cluster. Four genes have a high betweenness and three of them are hubs and have a high betweenness: SSR4, FTH1 and MGP. The specificity of this cluster is to be related to genes tightly transcriptionally regulated by the same complex of transcription factors. For example, MGP, MYH2, BMPR2, STC1, FTH1 and TPM3 expressions are regulated by NFAT (nuclear factor of activated T-cells). The gene transcription leads to protein synthesis, which is the biological function identified by IPA for this cluster (84% of the eligible genes are involved in protein synthesis and muscle development). The color and font meanings are given in Figure 8. (TIFF) [file pone.0060045.s005.tif]

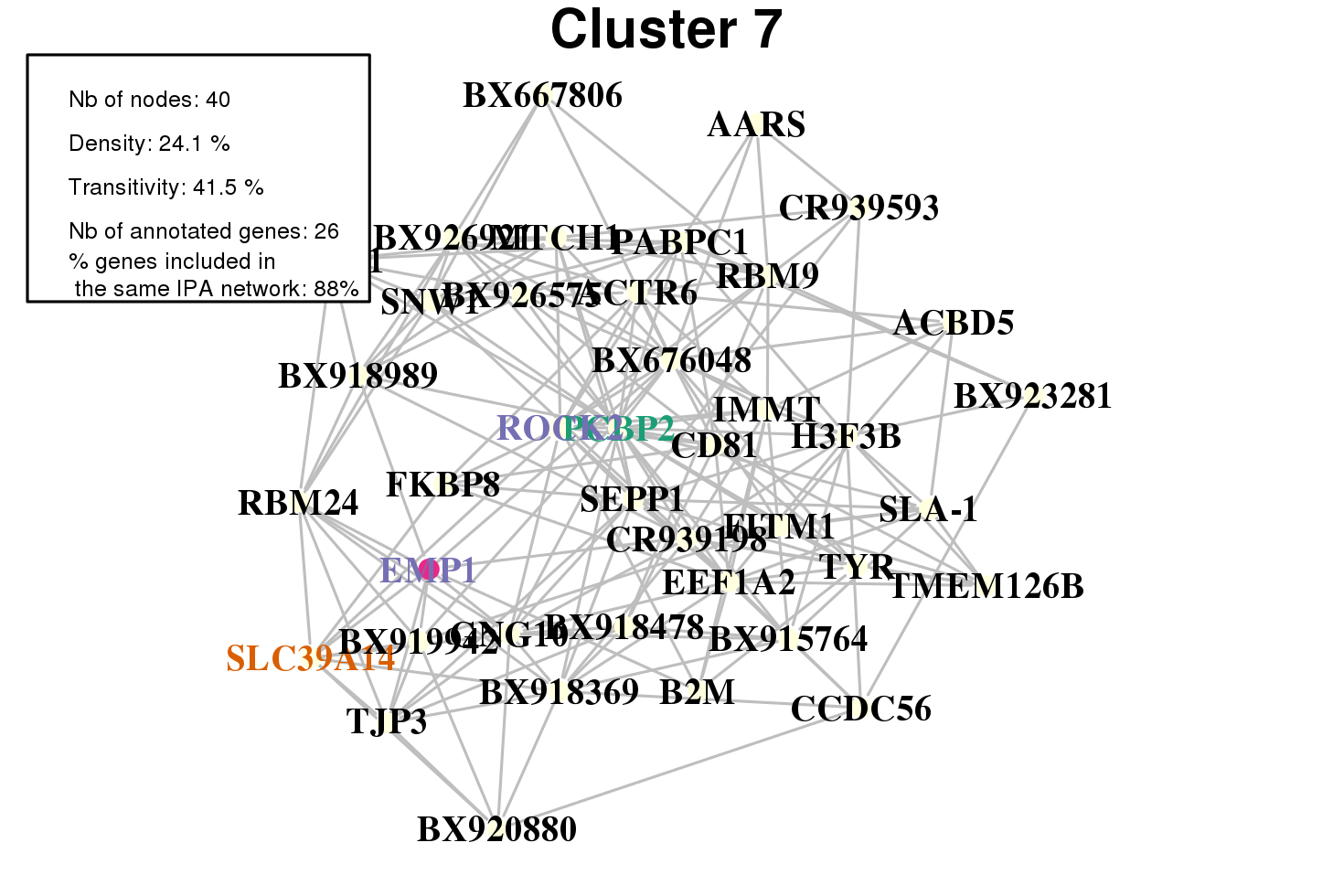

Supplement: Figure S6 — Cluster 7. This cluster seems to be organized around ROCK2 (Rho-associated coiled-coil forming kinase 2) and PCPB2 (poly(rC) binding protein 2). ROCK2 has high betweenness and was identified as being involved in cell death process (Shi and Wei, 2007). Cell death corresponds to the main biological function of this cluster identified by IPA, with 88% of the eligible genes. PCPB2 is a hub in this cluster while PCPB2 is also a cis-eQTL. This suggests a possible central role genetically controlled at the PCPB2 locus itself. PCPB2 with AARS, PABPC1 and SNW1 are involved in the gene expression regulation, especially via RNA spicing (Genecodis analysis, [65]). SLC39A14 (solute carrier family 39 (zinc transporter), member 14) is both a hub and a high betweenness node. Zinc is an essential cofactor for hundreds of enzymes. It is involved in protein, nucleic acid, carbohydrate and lipid metabolism, as well as in the control of gene transcription, growth, development and differentiation. The Zn transporter SLC39A14 controls the G-protein coupled receptor (GPCR)-mediated signaling [66]. Cell signaling through GPCR (G protein-coupled receptors) plays a central role in mediating multiple signaling pathways. The color and font meanings are given in Figure 8. (TIFF) [file pone.0060045.s006.tif]
